# Effectiveness of Housing First with Intensive Case Management in an Ethnically Diverse Sample of Homeless Adults with Mental Illness: A Randomized Controlled Trial

**DOI:** 10.1371/journal.pone.0130281

**Published:** 2015-07-15

**Authors:** Vicky Stergiopoulos, Agnes Gozdzik, Vachan Misir, Anna Skosireva, Jo Connelly, Aseefa Sarang, Adam Whisler, Stephen W. Hwang, Patricia O’Campo, Kwame McKenzie

**Affiliations:** 1 Centre for Research on Inner City Health, Li Ka Shing Knowledge Institute, St. Michael’s Hospital, Toronto, Ontario, Canada; 2 Department of Psychiatry, University of Toronto, Toronto, Ontario, Canada; 3 Toronto North Support Services, Toronto, Ontario, Canada; 4 Across Boundaries, Toronto, Ontario, Canada; 5 Division of General Internal Medicine, Department of Medicine, University of Toronto, Toronto, Ontario, Canada; 6 Dalla Lana School of Public Health, University of Toronto, Toronto, Ontario, Canada; 7 Health Services and Health Equity Research, Centre for Addiction and Mental Health, Toronto, Ontario, Canada; Nagoya University Graduate School of Medicine, JAPAN

## Abstract

Housing First (HF) is being widely disseminated in efforts to end homelessness among homeless adults with psychiatric disabilities. This study evaluates the effectiveness of HF with Intensive Case Management (ICM) among ethnically diverse homeless adults in an urban setting. 378 participants were randomized to HF with ICM or treatment-as-usual (TAU) in Toronto (Canada), and followed for 24 months. Measures of effectiveness included housing stability, physical (EQ5D-VAS) and mental (CSI, GAIN-SS) health, social functioning (MCAS), quality of life (QoLI20), and health service use. Two-thirds of the sample (63%) was from racialized groups and half (50%) were born outside Canada. Over the 24 months of follow-up, HF participants spent a significantly greater percentage of time in stable residences compared to TAU participants (75.1% 95% CI 70.5 to 79.7 vs. 39.3% 95% CI 34.3 to 44.2, respectively). Similarly, community functioning (MCAS) improved significantly from baseline in HF compared to TAU participants (change in mean difference = +1.67 95% CI 0.04 to 3.30). There was a significant reduction in the number of days spent experiencing alcohol problems among the HF compared to TAU participants at 24 months (ratio of rate ratios = 0.47 95% CI 0.22 to 0.99) relative to baseline, a reduction of 53%. Although the number of emergency department visits and days in hospital over 24 months did not differ significantly between HF and TAU participants, fewer HF participants compared to TAU participants had 1 or more hospitalizations during this period (70.4% vs. 81.1%, respectively; P=0.044). Compared to non-racialized HF participants, racialized HF participants saw an increase in the amount of money spent on alcohol (change in mean difference = $112.90 95% CI 5.84 to 219.96) and a reduction in physical community integration (ratio of rate ratios = 0.67 95% CI 0.47 to 0.96) from baseline to 24 months. Secondary analyses found a significant reduction in the number of days experiencing problems due to alcohol use among foreign-born (vs. Canadian-born) HF participants at 24 months (ratio of rate ratios = 0.19 95% 0.04 to 0.88), relative to baseline. Compared to usual care, HF with ICM can improve housing stability and community functioning and reduce the days of alcohol related problems in an ethnically diverse sample of homeless adults with mental illness within 2-years.

**Trial Registration:**

Controlled-Trials.com ISRCTN42520374.

## Introduction

Homelessness is a significant social problem in Toronto, Canada’s largest and most ethnically diverse urban center, where approximately 29,000 individuals use shelters each year and roughly 5,000 people are homeless on any given night [1, 2].

To address the pervasive problem of homelessness among individuals experiencing mental illness, in 2009, the Mental Health Commission of Canada (MHCC) launched the At Home/Chez Soi (AH/CS) research demonstration project, a four-year multi-site randomized controlled trial of Housing First (HF) [3]. Developed in New York City by *Pathways to Housing* [4], HF provides immediate access to independent housing and mental health supports, typically Assertive Community Treatment (ACT) [4, 5]. Previous studies from the US have shown that the HF model can improve housing and other outcomes among homeless individuals with mental illness [5–18]; however the AH/CS project is the first to evaluate the HF approach outside of the U.S. service context and with both ACT and Intensive Case Management (ICM) levels of support.

HF with ICM may offer a less costly intervention appropriate for a broader sample of homeless individuals with mental illness who do not require the intensity of ACT services. Recent reviews of interventions for homeless adults have shown that ICM either alone or in conjunction with housing can result in several positive outcomes, including improvement of psychiatric symptoms, decrease of substance use, and reduced in-patient service use [19, 20]. The combined approach, HF with ICM support, has been employed since 2005 by *Streets to Homes* (a City of Toronto program serving street dwelling individuals) [6]. While HF with ICM support has previously been evaluated by a randomized trial among homeless veterans in the US [11, 21], it has not been rigorously examined outside the US context and not with an ethnically diverse population. The Toronto site of the AH/CS project allows for the opportunity to evaluate the effectiveness of HF with ICM via a randomized controlled trial design in an ethnically diverse homeless population, who may experience additional barriers in accessing appropriate and timely housing and mental health care, within a system of universal access to health care [22–25].

Nearly half of Toronto residents are foreign born and 47% are from racialized groups. Racialized groups are defined as populations other than White and Aboriginal peoples, whose perceived race has shaped their experience through the process of racialization [26]. These groups were previously identified as “visible minority” or “ethno-racial” by Statistics Canada [27, 28]. Among the Toronto homeless population, a recent study of shelter or meal program users reported that nearly half (45%) identified as belonging to a non-White ethnic group [29]. The City of Toronto has recognized that both racialized and immigrant groups are at high risk of homelessness [30]. Recent studies have confirmed that racialized homeless adults living in Toronto face multiple levels of discrimination and stigma [31, 32].

Studies from Canada, US, UK and Australia suggest that both mental health service use patterns and access to care differ when immigrant and racialized groups are compared to the host White populations [22, 24, 33–39]. However, a recent review reports that while studies have shown rates of mental illness vary by national origin among Canadian residents, the current literature is limited in scope, study location (most studies were conducted in three major urban areas) and lacks information on non-immigrant racialized Canadians [40]. There is a growing literature of the needs for mental health services for homeless populations [20, 41]; however few studies on the mental health of homeless groups also investigate ethnic diversity [42]. It is unclear what the specific service needs are for ethnically diverse populations, or whether there are specific problems with access to or effectiveness of interventions.

Employing the framework that policy needs to be informed not only by which programs work best, but what programs work best for whom and in what contexts [43], the goal of this paper is to evaluate the effectiveness of HF with ICM (HF-ICM) at 24 months post-randomization in an ethnically diverse sample of homeless individuals with mental illness who reside in a service-rich urban center. The primary outcome of this study is the percentage of days stably housed. Secondary outcomes include physical and mental health, social functioning, quality of life, and health service use. We also explore the effect of ethnicity and immigration status on outcomes.

## Methods

### Sampling and Recruitment

Study participants met three inclusion criteria: (1) were ≥18 years old, (2) were absolutely homeless (defined as having no fixed place to stay for at least the past 7 nights, with little likelihood of finding a place in the upcoming month) or precariously housed (housed in single room occupancy, rooming house, or hotel/motel as a primary residence AND having a history of 2 or more episodes of absolute homelessness in the past year), and (3) had a serious mental disorder with or without co-existing substance use disorder [3]. The presence of a serious mental disorder was determined by the DSM-IV [44] criteria in the Mini International Neuropsychiatric Interview 6.0 (MINI) [45] by research staff; eligible diagnosis included at least one of: i) major depressive episode; ii) manic or hypomanic episode; iii) mood disorder with psychotic features; iv) panic disorder; v) post-traumatic stress disorder; and vi) psychotic disorder. Individuals were excluded from the study if they were: i) relatively homeless (individuals who inhabit spaces that do not meet the basic health and safety standards, such as living in overcrowded or hazardous conditions), ii) had illegal status in Canada, or iii) were currently receiving ACT or ICM [3].

Recruitment lasted from October 2009 to June 2011. A targeted recruitment strategy was employed to ensure that the study sample adequately represented the population of homeless people living in Toronto, based on a 2009 census [46]. Participants from ethno-racial minority groups were oversampled among the moderate needs group, to address the research questions. A study intake coordinator assessed eligibility for the study for all participants. More details on sampling, recruitment and eligibility criteria for the AH/CS study can be found elsewhere [3].

### Ethics statement

The study was approved by the Research Ethics Board of St. Michael’s Hospital in Toronto, and was registered with the International Standard Randomized Control Trial Number (ISRCTN 42520374). All study participants provided written informed consent.

### Study design

The AH/CS study is a randomized controlled trial of HF, and has been described previously [3].

Participants were considered “racialized” if they self-identified as being non-Caucasian in race or non-White in color, excluding Aboriginal peoples [27]. This definition was operationalized to include the following ethnic or cultural identities: East Asian (e.g. China, Japan, Korea), South Asian (e.g. India, Pakistan, Sri Lanka), South East Asian (e.g. Malaysia, Philippines, Vietnam), Black African (e.g. Ghana, Kenya, Somalia), Black Canadian/American, Black Caribbean Region (e.g. Jamaica, Trinidad, Tobago), Latin American (e.g. Argentina, Chile, Costa Rica), Indian-Caribbean (e.g. Guyana with origins in India), Middle Eastern (e.g. Egypt, Iran, Israel, Palestine) or Mixed background (that included at least one of the ethnic groups listed above). Participants who self-identified as Aboriginal, White (European or Canadian), Mixed ethnicity (that did not include any of ethnic groups listed above) or other ethnicity were considered “non-racialized”.

Prior to randomization, all eligible participants were stratified according to the severity of their mental health problems into High Needs or Moderate Needs groups for mental health services. The criteria for the need level groups included the results of MINI and the Multnomah Community Ability Scale (MCAS), presence of a co-morbid substance use disorder, and prior hospitalizations and incarcerations [47, 48]. A stratification algorithm and operational definitions for High vs. Moderate Needs groups in the AH/CS study are described in detail elsewhere [3].

Randomization was performed via adaptive randomization procedures [3, 49]. Adaptive randomization can ensure better balance between groups in small and moderate sized studies than strict randomization, by continually adjusting the probability of assignment to either group, depending on existing group assignment [3, 49]. Participants were immediately notified of their treatment allocation via a laptop connected to the study data management centre during a research interview [3]. Due to the nature of the intervention, the study was not blinded. The allocation algorithm was concealed from both the participants and the research staff.

**Fig 1:**
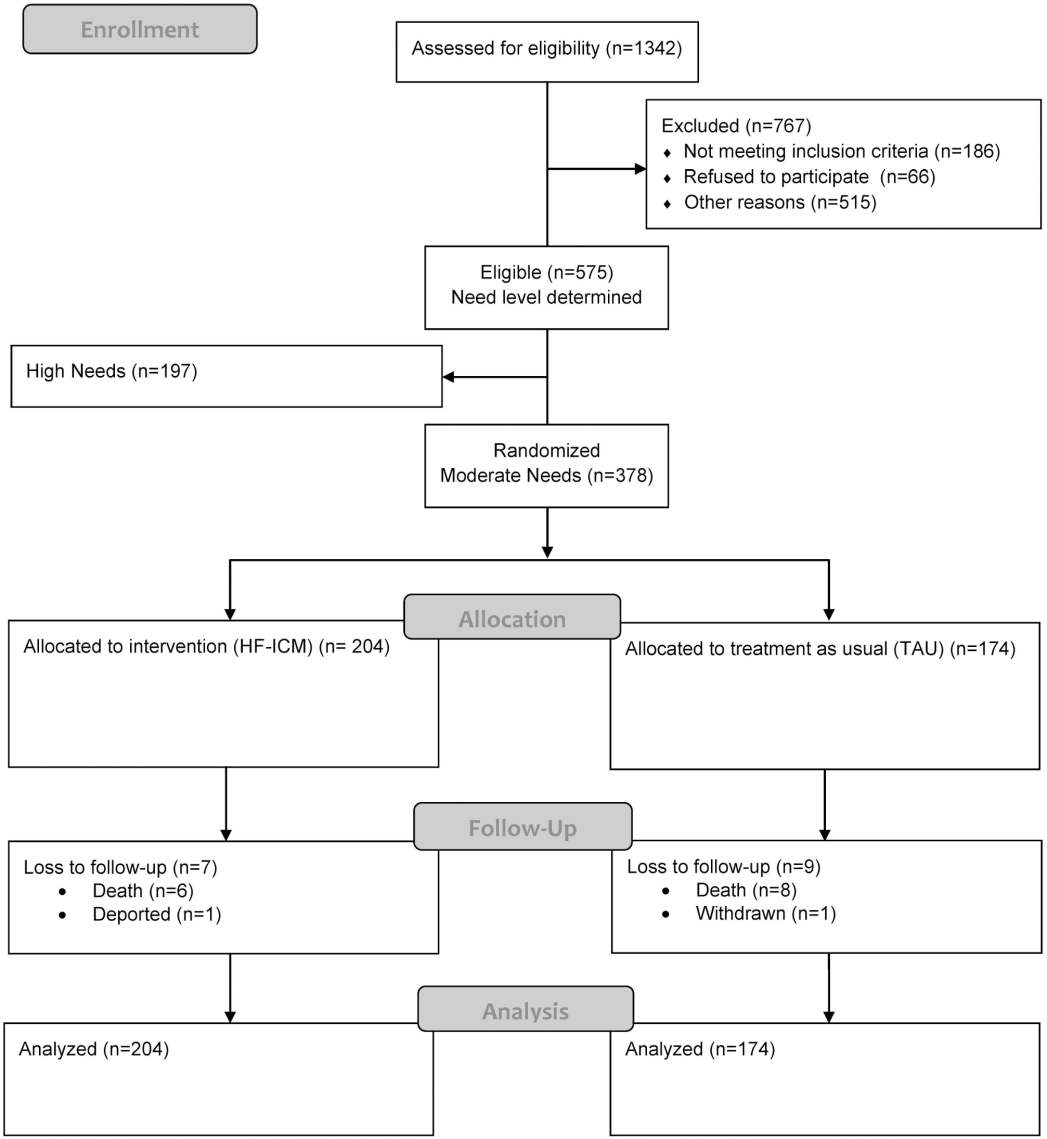
Participant flow through the study.

A diverse sample of moderate needs participants (N = 378) was randomized to treatment-as-usual (TAU) or to one of two HF-ICM teams. Both service teams offered ICM using a recovery oriented, trauma informed approach and harm reduction principles. One of the teams in addition had extensive experience offering services to racialized groups using an anti-racism and anti-oppression framework and only accepted racialized participants. Although White and Aboriginal participants randomized to the intervention were assigned to the non-specialized team, racialized participants randomized to the intervention were assigned to either of the two ICM teams [50]. Service teams at all sites underwent repeated fidelity assessments to ensure adherence to HF principles and standards [51]. See Fig 1 for participant study flow diagram and below for program description. For the purpose of this analysis, we examined the effectiveness of the intervention in our total sample of moderate needs participants.

### Intervention and control groups

Participants in the intervention groups were assigned to a case manager who worked with them to develop an individualized service plan based on their recovery goals, which could include supportive counselling, resource brokerage (for housing, education, employment, health, and legal issues), advocacy, skills teaching as well as crisis intervention support. The participant-to-staff ratio was 17:1. HF-ICM was provided for the duration of the follow-up period. Housing was provided in independent scattered-site housing. Housing expenses were covered by a study rent allowance of $600 CAD (paid directly to the landlord) and up to 30% of the participant monthly income. Participants were not required to accept or adhere to psychiatric or other treatment programs and did not have any restrictions regarding substance use. Services were participant-driven and not linked to tenancy.

Individuals randomized to the TAU group were able to access a variety of traditional housing programs and community services available in the city of Toronto, which include services geared to the homeless population (drop-in centers, emergency shelters, meal programs, street outreach services, supportive and alternative housing), in addition to several mental health services available to both homeless and housed individuals (inpatient and outpatient services, case management, ACT, court support services, crisis programs, and ethno-racial focused agencies). TAU participants were provided with information on these existing services.

Further details on the stratification algorithm, randomization procedures and intervention content of the Toronto site AH/CS study are described elsewhere [52].

### Data collection

Data were collected via structured, face-to-face laptop computer-assisted interviews and entered into a secured central database via wireless technology. Follow-up interviews were conducted every three months, with longer interviews taking place at baseline, 6-, 12-, 18- and 24-months. Interviews took place in various locations, based on participant choice, including in the community (e.g. coffee shops), at the participants’ residence, at the study office or local hospitals. To reduce attrition rates and maintain contacts, brief monthly call-in updates and interviews were conducted. Participants received financial compensation for these updates and interviews.

### Outcome measures

The primary study outcome was housing stability which was assessed using the Residential Time Line Follow-back (RTLFB) inventory [53]. For each participant, the percentage of days spent stably housed during the 24 month study period was calculated from the recorded number of days spent in stable housing, divided by the total number of days accounted during the 24 month study period. In addition, the rate of hospitalization was also assessed from the RTLFB by examining the number of days spent overnight in a hospital during the time period for which residential data was available during the follow up period.

The study had several secondary outcome measures that covered a variety of domains, including 1) physical and mental health; 2) social functioning and quality of life; and 3) health service use.

#### Physical and mental health

Physical and mental health were assessed using the EuroQuol 5 Dimensions Visual Analogue Scale (EQ5D-VAS) [54] and the 14-item modified Colorado Symptom Index (CSI) [55], respectively. Past-month frequency of substance use problems was evaluated using the Substance Disorder Screener (SDScr) of the GAIN Short Screener (GAIN-SS) [56]. Four additional questions, which pertained to the number of days experiencing problems due to alcohol or substance use in the past month, and the amount of money spent on alcohol or substances in the past month, respectively, were also evaluated.

#### Social functioning and quality of life

Social functioning was assessed using the physical and psychological subscales of the Community Integration Scale (CIS) [57], in addition to the summary score of the Multnomah Community Ability Scale (MCAS) [47, 48, 58]. Quality of life was assessed using the summary score from the Quality of Life Index (QoLI-20) [59, 60].

#### Health service use

Two questions from a Health Service and Justice Service Use (HSJSU) Questionnaire [3] were examined: i) “In the past 6 months, have you been to a hospital emergency room?”; ii) “Approximately how many emergency room visits did you have in total?”.

### Statistical analyses

We chose the MCAS (which measures multiple dimensions of community functioning) as the outcome variable with which to establish a clinically meaningful effect size. Previous studies employing the MCAS have reported a standard deviation of approximately 9 [58, 61]; therefore we sought a difference of approximately 4.5 points of the MCAS for a moderate effect size in our sample. With an assumed attrition rate of 25%, we calculated that a sample size of 75 participants was necessary in each arm, providing 86% power at an α of 0.05 in 2-sided statistical tests.

All analyses were performed on an intention-to-treat basis with a significance level set at α = 0.05 using SAS version 9.4. The primary outcome, percent of days stably housed during the 24 month follow-up period, is the number of days stably housed during this period divided by the total number of days accounted for during the 24-month period, was calculated only at one time point (24 months), and analyzed by fitting a linear regression under the mixed model framework which assessed the main effect of treatment group (HF-ICM vs. TAU) and ethnicity (racialized or not), as well as the potential treatment group x ethnicity interaction and estimated the adjusted least square means which represent the marginal means for each main effect category. The number of days hospitalized was similarly analyzed by fitting a zero inflated negative binomial model that examined the main effect of treatment group and ethnicity, in addition to their interactions, and generated: 1) a rate ratio of the number of days spent overnight in a hospital over the total number of days for which residential data was available (for individuals who had at least 1 or more hospitalizations); and 2) the probability of having excessive zero values. In these analyses, the effectiveness of the intervention was assessed on the basis of the treatment effect.

All other secondary outcomes were measured at multiple time points and analyses were conducted using linear mixed models (PROC MIXED) for continuous outcomes and generalized linear models (PROC GENMOD/PROC GLIMMIX) for count variables. These models examined the main effects of time (baseline, 6-, 12-, 18- and 24-months), treatment group (HF-ICM vs. TAU), and ethnicity (racialized or not), in addition to the two-way interactions between the main effects. In these analyses, baseline values were used as a reference time point for all comparisons at subsequent time points (6-, 12-, 18- and 24-months) and the TAU group was used as a reference treatment group. The effectiveness of the intervention in these models was assessed on the basis of the time x treatment group interaction. For continuous outcomes, the time x treatment group interaction examined the change in the mean from baseline to a subsequent follow-up visit (6-, 12-, 18- and 24-months) for the HF-ICM group compared to the TAU group. For count outcomes, the time x treatment group interaction evaluated the ratio of rate ratios for each post-baseline visit (e.g. rate ratio at follow-up visit relative to baseline in the HF-ICM group divided by the rate ratio at follow-up visit relative to baseline in the TAU group). To investigate the effect of ethnicity on outcomes, a subsequent model examined the three-way interaction (time x treatment group x ethnicity), but only for those variables for which the main effect of ethnicity was significant (α<0.05).

In addition, we performed additional analyses to investigate the role of immigrant status in outcomes over time. Because a large number of ethno-racial participants were immigrants (which we defined as individuals who were born in countries other than Canada), we also wanted to examine if immigrant status rather than ethnicity had an effect on study outcomes. To do so, we re-analyzed all of the data using immigrant status (yes or no) in place of ethnicity in all of the analyses described above. For secondary outcomes measured at more than one time point, analytic models investigated both the two way interactions between main effects of time, treatment group, and immigrant status, as well as the three-way interaction (time x treatment group x immigrant status).

An unstructured covariance matrix was considered in all models.

### Missing data analysis

Prior to analyses, a strategy to perform multiple imputation using the sequential regression multivariate imputation approach was established, due to missing data that resulted from non-participation, withdrawal, and participant refusal to answer items or entire instruments during interviews [62, 63]. This multiple imputation method allows for efficient imputation by fitting a model to each variable, conditional on all others, and imputing one variable at a time [64, 65]. The multiple imputation model included outcomes at baseline, 6-, 12-, 18- and 24-months and was informed by participant gender, age at enrollment, ethnicity, and Aboriginal status. Forty imputations were stratified by treatment arm, a strategy needed since our analysis models would consider the interactions between treatment group and time and between treatment group and ethno-racial status [66, 67]. Imputed datasets were implemented using the *mi impute chained* command in STATA v. 13 (StataCorp LP) and results were combined using PROC MIANALYZE in SAS 9.4 (SAS Institute).

Due to the complexity of the RTLFB instrument, and the low level of missing data (5.0%), no imputation was performed for the two outcomes variables generated from it: days stably housed and rate of hospitalization.

## Results

### Sample description

Fig 1 shows the flow diagram of participants through the study. In total, 378 participants were randomized to either HF-ICM treatment (N = 204) or TAU group (N = 174). At 24 months post-randomization, 16 participants were lost to follow-up, 14 resulting from deaths and the others from withdrawal and deportation. Data from all participants, up until the point of their loss to follow-up were included in all analyses. The proportion of active participants who completed follow-up interviews at 12 and 24 months was 92% (343/374) and 85% (306/362), respectively.

### Baseline characteristics

Baseline characteristics of study participants are shown in Table 1. Study participants were primarily in their 40’s (32%), male (68%), native English speakers (60%), racialized (63%), single never-married (68%), and had no children (70%). Half of our participants had been born outside of Canada (50%). The majority of participants had been absolutely homeless at study start (92%), with a mean total length of homelessness of 4.7 ± 5.7 years. Almost all (95%) were unemployed, with half (47%) having completed less than a high school education. Most common diagnoses based on the MINI included substance dependence or abuse (46%), major depression (45%), alcohol dependence or abuse (40%), post-traumatic stress disorder (29%) and psychotic disorder (26%). Nearly 70% of participants reported suicidal ideation. HF-ICM and TAU group participants did not differ in any of the baseline characteristics examined.

**Table 1:** Baseline sample characteristics.

		Full Sample,^1^ (N = 378)	Housing First with ICM, (N = 204)	Treatment As Usual, (N = 174)	P Value
Age, N (%)					0.861
	<30	92 (24.3)	51 (25.0)	41 (23.6)	
	30–39	88 (23.3)	47 (23.0)	41 (23.6)	
	40–49	120 (31.7)	67 (32.8)	53 (30.5)	
	≥50	78 (20.6)	39 (19.1)	39 (22.4)	
Gender, N (%)^2^					
	Female	119 (32.2)	65 (32.0)	54 (32.3)	0.948
	Male	251 (67.8)	138 (68.0)	113 (67.7)	
Country of Birth, N (%)					
	Canada	191 (50.5)	101 (49.5)	90 (51.7)	0.668
	Other	187 (49.5)	103 (50.5)	84 (48.3)	
Native Language, N (%)					
	English	227 (60.1)	123 (60.3)	104 (59.8)	0.917
	Other	151 (39.9)	81(39.7)	70 (40.2)	
Ethnic or Cultural Identity, N (%)					
	Racialized^3^	237 (62.7)	135 (66.2)	102 (58.6)	0.13
	Non Racialized^4^	141 (37.3)	69 (33.8)	72 (41.4)	
Marital Status N (%)					
	Divorced/Separated/Widowed	108 (28.6)	57 (28.1)	51 (29.3)	0.943
	Married/Cohabitating With Partner	14 (3.7)	8 (3.9)	6 (3.4)	
	Single, Never Married	255 (67.6)	138 (68.0)	117 (67.2)	
Number of Children, N (%)					
	0	264 (70.2)	139 (68.1)	125 (72.7)	0.260
	1	61 (16.2)	38 (18.6)	23 (13.4)	
	2	33 (8.8)	15 (7.4)	18 (10.5)	
	≥3	18 (4.8)	12 (5.9)	6 (3.5)	
Current Status, N (%)					
	Absolutely Homeless	347 (91.8)	186 (91.2)	161 (92.5)	0.633
	Precariously Housed	31 (8.2)	18 (8.8)	13 (7.5)	
Total Length of Homelessness, Mean y (SD)^5^		4.69 ± 5.71	4.55 ± 5.63	4.86 ± 5.82	0.512
Longest Period of Homelessness, Mean y (SD)^6^		2.54 ± 3.89	2.50 ± 4.12	2.58 ± 3.61	0.455
Education History, N (%)					
	< High School	178 (47.2)	103 (50.5)	75 (43.4)	0.346
	Completed High School	70 (18.6)	34 (16.7)	36 (20.8)	
	Some Post-Secondary School	129 (34.2)	67 (32.8)	62 (35.8)	
Employment Status, N (%)					
	Unemployed	358 (94.7)	193 (94.6)	165 (94.8)	0.924
	Employed	20 (5.3)	11 (5.4)	9 (5.2)	
MCAS, score (SD)^7^		65.2 ± 3.39	65.2 ± 3.25	65.3 ± 3.55	0.479
MINI Results, N (%)^8^					
	Depressive Episode	171 (45.2)	92 (45.1)	79 (45.4)	0.953
	Manic or Hypomanic Episode	41 (10.8)	25 (12.3)	16 (9.2)	0.340
	Post-Traumatic Stress Disorder	109 (28.8)	61 (29.9)	48 (27.6)	0.620
	Panic Disorder	72 (19.0)	38 (18.6)	34 (19.5)	0.822
	Mood Disorder with Psychotic Features	71 (18.8)	38 (18.6)	33 (19.0)	0.933
	Psychotic Disorder	99 (26.2)	55 (27.0)	44 (25.3)	0.712
	Alcohol Dependence or Abuse	152 (40.2)	78 (38.2)	74 (42.5)	0.396
	Substance Dependence or Abuse	175 (46.3)	93 (45.6)	82 (47.1)	0.765
	Suicidality^9^	261 (69.0)	135 (66.2)	126 (72.4)	0.191

*P* Values correspond to chi-square tests for categorical variables and Mann-Whitney tests for continuous variables.

^1^For the total sample, percentages shown were calculated as proportion of the total sample (N = 378) except for the following variables which had missing values: Marital status (N = 1); Number of children (N = 2); Total length of homelessness (N = 5); Longest period of homelessness (N = 3); Education History (N = 1). For group value calculations, percentages are calculated out of the total available data (excluding missing); therefore column totals for each variable add up to 100%. However, for questions with “yes/no” answers, only the proportion of individuals who indicated “yes” are provided.

^2^For Gender, individuals who self-identified as Other/Transgendered/Transsexual were not included (N = 8) due to small cell size

^3^ “Racialized” includes participants who indicated the following ethnic or cultural identities: East Asian (e.g. China, Japan, Korea), South Asian (e.g. India, Pakistan, Sri Lanka), South East Asian (e.g. Malaysia, Philippines, Vietnam), Black African (e.g. Ghana, Kenya, Somalia), Black Canadian/American, Black Caribbean Region (e.g. Jamaica, Trinidad, Tobago), Latin American (e.g. Argentina, Chile, Costa Rica), Indian-Caribbean (e.g. Guyana with origins in India), Middle Eastern (e.g. Egypt, Iran, Israel, Palestine) or Mixed background (that included at least one of the ethnic groups listed above).

^4^ The “Non Racialized” category includes participants who indicated the following ethnicities: White-Canada, White-Europe and Other and includes participants who self-identified as Aboriginal (n = 18).

^5^ Median values (y) for Total Length of Homelessness: Full sample: 2.67, Housing First with ICM: 2.50, Treatment as Usual: 2.83.

^6^Median values (y) for Longest Period of Homelessness: Full sample: 1.0, Housing First with ICM: 1.0, Treatment as Usual: 1.0.

^7^Median values for MCAS score: Full sample: 65.0, Housing First with ICM: 65.0, Treatment As Usual: 65.0

^8^MINI Diagnoses all represent current diagnoses established at baseline.

^9^Suicidality was assessed as low, medium, or high; results here are shown with categories collapsed.

**Fig 2:**
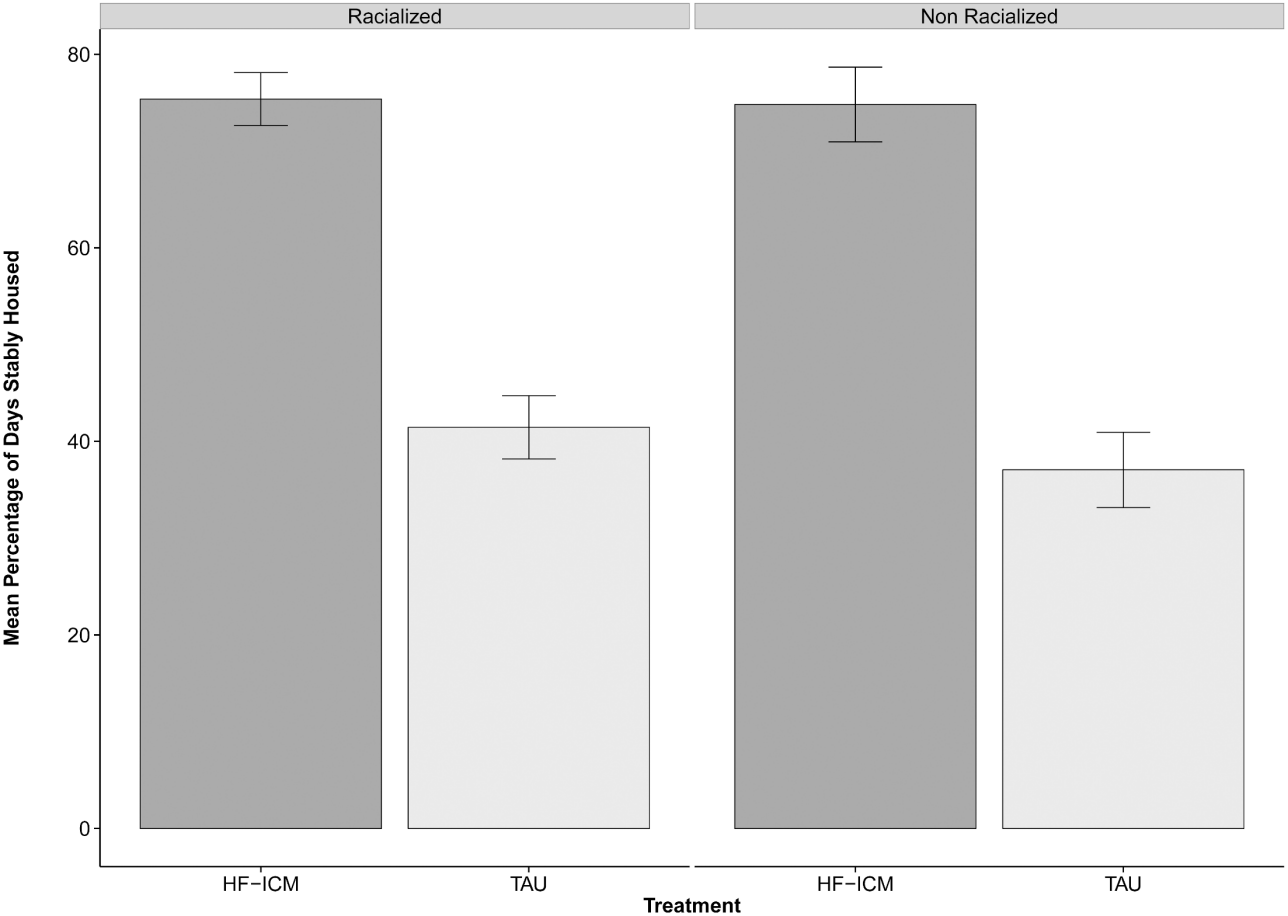
Primary study outcome, by treatment group and ethnicity. Values correspond to adjusted means and bars correspond to standard errors from linear regression model (using a mixed effect model framework) for the percentage of days stably housed over 24 month follow-up period for HF-ICM and TAU groups among participants, by ethnicity.

**Fig 3:**
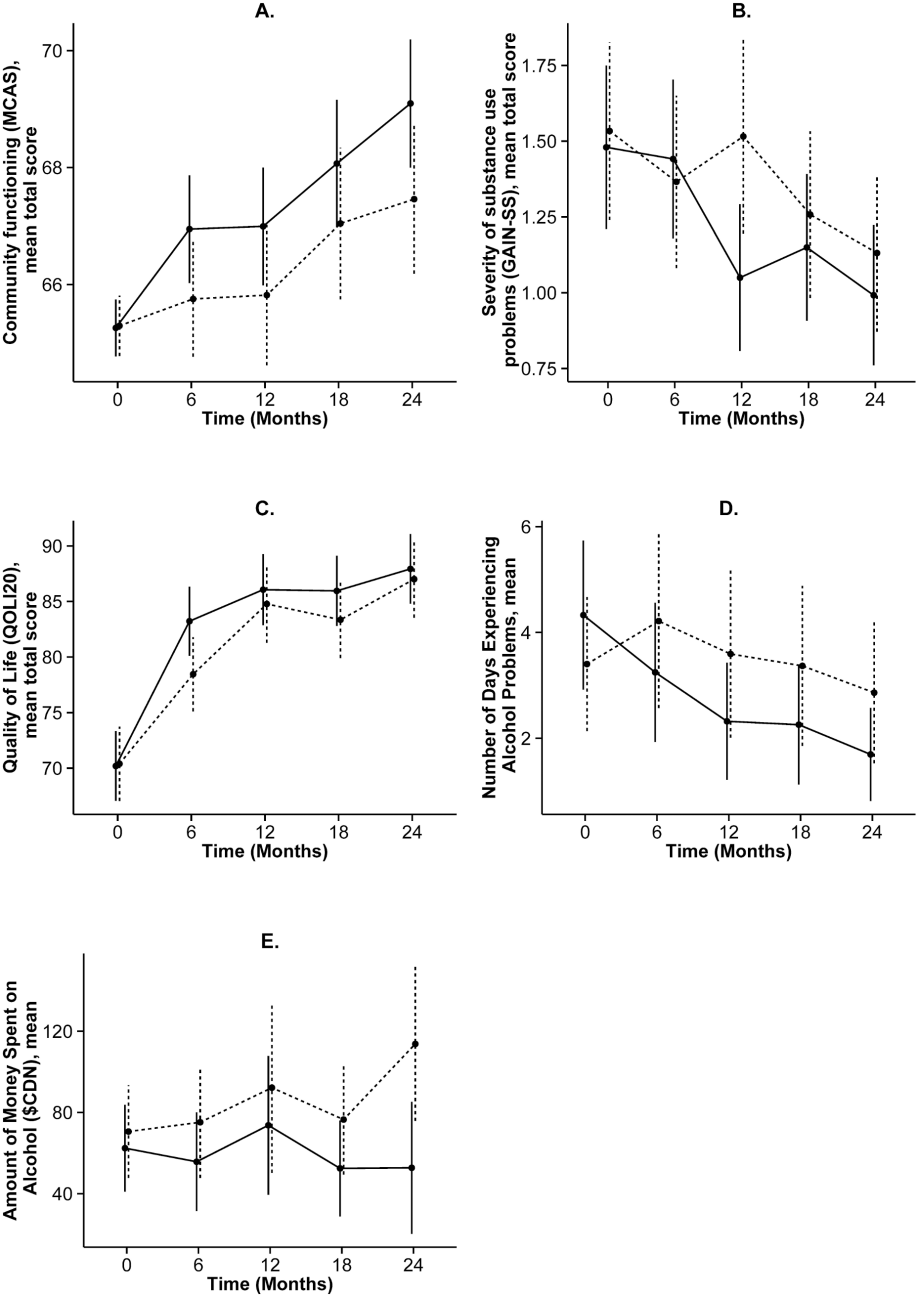
Select secondary outcomes, mean (95% CI), by treatment group over time. Solid and dashed lines indicate the HF-ICM and TAU groups, respectively.

Fig 2 shows the primary outcome variable (housing stability), which was measured over the entire 24 month period, while Fig 3 shows the change over time in select secondary outcome variables, which were analyzed at five study time points. S1 Table and S2 Table provide more detailed results from these analyses.

### Housing stability

During the study period, participants in the HF-ICM group spent a significantly higher percentage of time in stable residences compared to those in the TAU group (75.1 95% CI 70.5 to 79.7 vs. 39.3 95% CI 34.3 to 44.2, respectively]. Fig 2 shows the proportion of time spent stably housed by treatment group and ethnicity.

### Physical and mental health

The intervention did not result in any significant treatment x time interactions in either physical health (EQ-5D VAS) or mental illness symptomatology (CSI) at either 12 or 24 months (S1 Table and S2 Table).

Severity of substance use related problems, as assessed by the GAIN-SS, were significantly reduced in the HF-ICM group compared to the TAU group from baseline to 12 months (ratio of rate ratios = 0.72 95% CI 0.53 to 0.98) corresponding to a reduction of 28%, but this difference was not statistically significant at 24 months (ratio of rate ratios = 0.91 95% CI 0.65 to 1.28) (Fig 3). In analyses of the four additional substance use related questions, there was a significant reduction in the number of days spent experiencing alcohol problems among the HF-ICM compared to the TAU group at both 12 months (ratio of rate ratios = 0.51 95% CI 0.28 to 0.93) and 24 months (ratio of rate ratios = 0.47 95% CI 0.22 to 0.99) relative to baseline, corresponding to a reduction of 49% and 53%, respectively (Fig 3). In addition, the amount of money spent on alcohol the past 30 days showed a statistically significant reduction from baseline to 24-months in the HF-ICM compared to the TAU group (change in mean difference = -$52.86 95% CI -104.29 to -1.43). No significant group differences were observed for substance use, either in the number of days experiencing problems or the amount of money spent (S1 Table and S2 Table).

### Social functioning and quality of life

Community functioning (assessed by MCAS) improved significantly in the HF-ICM group compared to the TAU group at 24-months (change in mean difference = +1.67 95% 0.05 to 3.30) (Fig 3). Quality of life (QoLI-20) improved significantly in the HF-ICM compared to the TAU group from baseline to 6 months (change in mean difference = +4.97 95% CI 0.27 to 9.67), however, this difference did not remain significant at later study visits. Neither psychological nor physical community integration showed significant differences between the treatment groups from baseline to any of the follow-up time points (S1 Table and S2 Table).

### Health care utilization

The mean number of hospital days in the follow-up period, among participants who had at least one hospitalization, was similar for participants in the HF-ICM and TAU groups (19.4 95% CI 12.0 to 31.4 and 10.4 95% CI 5.69 to 19.0, respectively). The probability of having at least one hospitalization differed significantly between the two groups, with participants in the HF-ICM group (70.4%) being less likely to be hospitalized than those in the TAU groups (81.1%; P = 0.044).

No statistically significant differences were observed in the number of emergency department visits between groups from baseline to any other study time point (S2 Table).

### Outcome differences among racialized groups

Ethnicity had a significant main effect on physical health (EQ5D-VAS; P = 0.003), severity of substance use related problems (GAIN-SS; P<0.001), number of emergency department visits (P = 0.004), amount of money spent on alcohol (P<0.001) and drugs (P = 0.0003) and the number of days experiencing problems due to alcohol (P = 0.004) and drug (P = 0.0006) use. In all associations, greater improvement was observed among racialized participants, compared to non-racialized participants.

**Fig 4:**
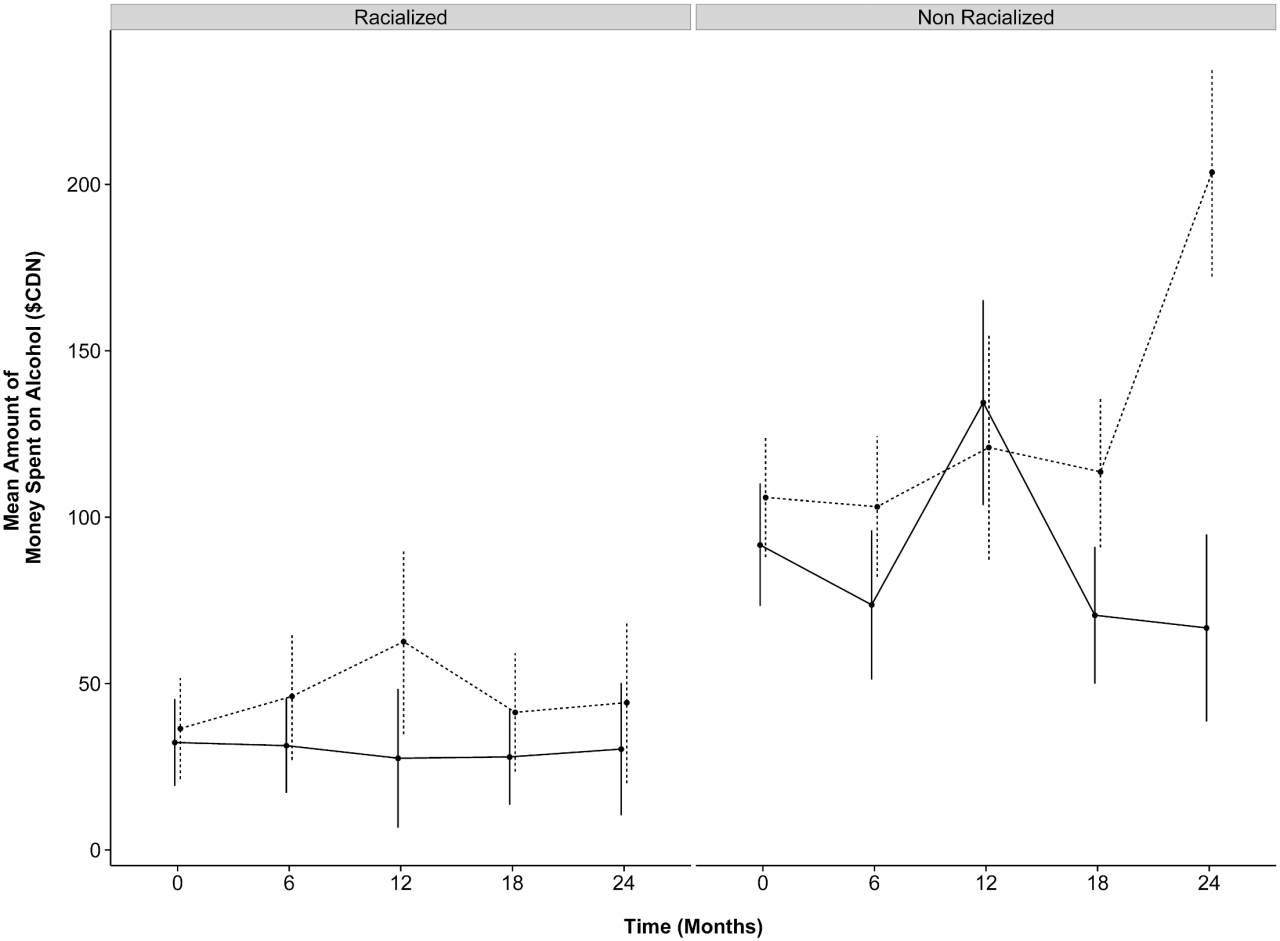
Mean (95% CI) amount spent on alcohol, by treatment group and ethnicity over time. Solid and dashed lines indicate the HF-ICM and TAU groups, respectively. Among racialized participants, HF-ICM participants decreased spending from $32.30 at baseline to $30.32 at 24 months, while TAU participants increased spending from $36.50 to $44.29 from baseline to 24 months, respectively. Among non-racialized participants, HF-ICM participants decreased spending from $91.69 at baseline to $66.73 at 24 months, while their TAU counterparts increased from $105.91 to $203.63 from baseline to 24-months, respectively. All values are in $CAD.

However, a statistically significant three-way interaction (time x treatment group x ethnicity) was only detected at 24 months relative to baseline for physical community integration and the amount of money spent on alcohol in the past 30 days. Physical community integration decreased significantly from baseline to 24 months among racialized participants in the HF-ICM group, compared to those who were not racialized (ratio of rate ratios = 0.67 95% CI 0.47 to 0.96), corresponding to decrease of 33%. Compared to participants who were not racialized, racialized participants in the HF-ICM compared to the TAU group spent more money on alcohol at 24 months relative to baseline (change in mean difference = + $112.90 95% CI 5.84 to 219.96) (Fig 4).

### Effect of immigrant status on outcomes variables

There were no changes in the primary outcome of housing stability when immigrant status rather than ethnicity was used as a covariate in the model: participants in the HF-ICM group spent a significantly higher percentage of time in stable residences compared to those in the TAU group (75.2% vs. 39.5%; P<0.001).

In models of secondary outcomes using immigrant status as a covariate, only community functioning (MCAS) and number of days experiencing problems due to alcohol use showed a significant treatment group x time interaction from baseline to 24 months, both indicating greater improvement in the intervention group (change in mean difference = +1.67 95% CI 0.04 to 3.30 and ratio of rate ratios = 0.42 95% CI 0.20 to 0.88, respectively).

As in the principal analysis, the probability of having at least one hospitalization remained statistically lower in the HF-ICM (70.2%) compared to the TAU group (80.8%, P = 0.049), although the mean number of hospital days in the follow-up period among participants who had at least one hospitalization did not differ between groups (P = 0.060).

In these models, we also examined the main effect of immigrant status on all outcome measures and found significant associations with severity of substance use problems (GAIN-SS; P<0.001), number of emergency department visits (P = 0.02), number of arrests (P = 0.001), the amount of money spent on both alcohol (P = 0.0009) and drugs (P<0.0001), as well the number of days experiencing problems due to alcohol use (P = 0.0064) and drug use (P<0.0001); in all associations immigrants showed greater improvements compared to their non-immigrant counterparts.

In subsequent three way interaction models (time x treatment group x immigrant status), immigrants in the HF-ICM group saw a significant differential reduction from baseline in the number of days spent experiencing alcohol use problems due to the intervention at 24 months post-randomization (ratio of rate ratios = 0.19 95% 0.04 to 0.88, P = 0.034), and the number of days experiencing problems due to substance use at 12 months, compared to participants in the TAU group (ratio of rate ratios = 0.22 95% 0.05 to 0.95, P = 0.042).

## Discussion

HF-ICM resulted in significant improvements in housing stability, probability of hospitalization, community functioning, and a reduction in number of days experiencing problems due to and money spent on alcohol use in an ethnically diverse sample of homeless individuals with mental illness living in a large urban metropolis. Interestingly, none of the outcomes examined showed differential treatment outcomes by racialized ethnicity except physical community integration and the amount of money spent on alcohol, both of which worsened among racialized participants compared to those who were not racialized, at 24 months post-randomization. Furthermore, immigrants to Canada experienced a greater reduction in the number of days spent experiencing problems associated with alcohol use due to the intervention than their Canadian-born counterparts, although no other outcomes showed differences due to the intervention by immigrant status at 24 months post-randomization.

The effectiveness of the HF approach, in particular HF with ICM, has not previously been rigorously assessed in an ethnically diverse population composed of a large number of immigrants. In this study, two-thirds of our sample indicated non-White non-Aboriginal ethnicity and half were immigrants to Canada. A recent article has noted the lack of literature on the unique housing needs of individuals of Aboriginal or non-European ethnicity, particularly in Canada [42]. Our results indicate that HF-ICM can result in improvements in housing stability, probability of hospitalization, community functioning and outcomes related to alcohol use in both racialized and non-racialized groups in the Canadian setting.

The literature on the effectiveness of HF interventions in reducing emergency department use and hospitalizations is scarce. A secondary analysis of a randomized controlled trial of HF with ACT reported a reduction in the proportion of time spent hospitalized in the treatment group, compared to control group; however, this result was only observed when participant recruitment source (from street or from psychiatric hospital) was incorporated in a three-way interaction (time x treatment group x sample source) [13]. Although not a HF program, a randomized trial of an intervention which provided housing in conjunction with case management upon discharge from hospital to chronically homeless individuals led to a reduction in both emergency department visits and hospitalizations [68]. In our study, HF-ICM did not lead to reductions in self-reported emergency department use or number of hospitalizations, however, the proportion of participants who had 1 or more hospitalizations during the study period was significantly lower among the HF-ICM participants compared to their TAU counterparts. Future analysis of administrative data will provide more accurate estimates of the effect of the HF-ICM intervention on health care utilization in the AH/CS project.

Community functioning, using the MCAS as an assessment and evaluation tool, has previously been reported in the Community Mental Health Evaluation Initiative (CMHEI), a longitudinal multi-site evaluation of community mental health programs in the province of Ontario (Canada) [69]. A group of clients (N = 91) from CMHEI programs in Hamilton, Ottawa and Toronto were assessed using MCAS at baseline and 9 months: scores increased from 67.4 ± 12.4 to 70.2 ± 9.79, respectively [70]. The magnitude of improvement in this previous study (+3 units) is comparable to the participants receiving ICM in the current study (+4).

Despite the effectiveness of HF in improving housing and other outcomes, previous research suggests that alcohol and substance related problems are more refractory to the intervention [5, 71, 72]. A prior randomized controlled trial examining the effectiveness of HF with ACT to standard care observed no significant differences in self-reported alcohol or drug use between the two groups over the first two years of follow-up [5], findings that persisted at 48 months [71]. A recent study examining 2-year alcohol use trajectories among project-based HF participants (offered within a single housing project with on-site services) observed a decrease in both alcohol use and alcohol-related problems, however the study did not have a randomized design, and the intervention differed considerably in both housing and service provision from the current study [73]. Our findings demonstrate an improvement in the number of days with alcohol related problems, in addition to a reduction in the amount of money spent on alcohol at 24 months post-randomization. Of note, compared to non-racialized participants in the intervention group, spending on alcohol from baseline to 24 months post-randomization was increased among racialized participants. This result is largely driven by the greater decrease in spending on alcohol among non-racialized HF participants from baseline to 24 months compared to racialized HF participants, for whom the amount of money spent on alcohol remained relatively unchanged. When examining absolute values, however, racialized participants spent a substantially lower amount of the money on alcohol throughout the study, as can be seen in Fig 4. Interestingly, results from our additional analyses suggest that non-Canadian-born participants experienced greater improvements in alcohol and substance use related problems compared to their Canadian-born counterparts, a finding that merits further examination in future work. It is important to note that our study reports both on the severity and frequency of alcohol or drug related problems as well as frequency of days experience problems due to alcohol and substance use, to capture improvements within a harm reduction framework.

The perceived reduction in physical community integration among racialized participants in the HF-ICM group, compared to those who were not racialized, at study end compared to baseline, is concerning and points to the need to further examine the unique needs of racialized homeless people regarding social inclusion and physical community integration. Physical community integration measured participation in outside sports, recreation or community events, attending concerts or going to the movies, and meeting others at restaurants or coffee shops, domains that may need further exploration in future studies.

This study has some limitations. Firstly, the follow-up period is relatively short for individuals experiencing chronic homelessness and it is possible that some outcomes may need a longer period to show improvement. Secondly, the uniqueness of the ethnic diversity of Toronto may mean that our results may not apply to other urban centres; however, given that other cities will likely be less diverse, our results lend strong support to the effectiveness of this approach elsewhere. Thirdly, in a service-rich city like Toronto, the differences between the services received by some participants in the HF-ICM and TAU groups during the follow-up period may have been similar. Considerable resources exist within the city of Toronto that serves homeless people and/or people with mental illness, including several ACT and ICM teams, which may have contributed to the lack of further differences between the groups in other study outcomes. Finally, several variables were self-reported, including health care use, and are therefore potentially influenced by recall bias.

## Conclusions

HF with ICM leads to substantial and rapid improvement in housing stability in an ethnically diverse sample of homeless adults with mental illness. The intervention also leads to significant reductions in probability of hospitalization, community functioning and number of days experiencing alcohol related problems. Although future research will help further clarify the longer-term effects of this approach, our findings suggest that HF with ICM can be effective in a diverse population of homeless individuals experiencing mental illness that may face additional barriers to accessing and engaging in treatment.

## Supporting Information

S1 Table Estimated outcome values at each study visit, by treatment group. (DOCX)

S2 Table Treatment group differences at each study visit and changes from baseline at post-baseline visits. (DOCX)
