# Supplementary material for: Effectiveness of Housing First with Intensive Case Management in an Ethnically Diverse Sample of Homeless Adults with Mental Illness: A Randomized Controlled Trial
Source: PLoS One. 2015 Jul 15;10(7):e0130281. doi: 10.1371/journal.pone.0130281 (PMC4503775; doi:10.1371/journal.pone.0130281)
Supplement: S1 Table — (DOCX) [file pone.0130281.s001.docx]

**S1 Table: Estimated outcome values at each study visit, by treatment group.**

|  |  | Baseline | | 6 Months | | 12 Months | | 18 Months | | 24 Months | |
| --- | --- | --- | --- | --- | --- | --- | --- | --- | --- | --- | --- |
|  |  | HF-ICM | TAU | HF-ICM | TAU | HF-ICM | TAU | HF-ICM | TAU | HF-ICM | TAU |
| Physical and Mental Health |  |  |  |  |  |  |  |  |  |  |  |
|  | Health Status (EQ5D-VAS) | 59.3 (55.8 to 62.7) | 60.8 (57.2 to 64.4) | 63.8 (60.4 to 67.3) | 63.3 (59.4 to 67.2) | 64.7 (61.3 to 68.0) | 62.9 (59.0 to 66.8) | 63.7 (60.3 to 67.1) | 67.8 (63.8 to 71.7) | 64.9 (61.7 to 68.1) | 67.7 (64.0 to 71.3) |
|  | Mental Illness Symptomatology (CSI) | 40.3 (38.6 to 42.0) | 40.9 (39.0 to 42.7) | 35.0 (33.3 to 36.7) | 36.9 (35.1 to 38.7) | 33.3 (31.6 to 35.0) | 34.8 (32.8 to 36.8) | 32.5 (30.8 to 34.2) | 33.1 (31.3 to 35.0) | 31.8 (30.1 to 33.5) | 32.9 (31.0 to 34.8) |
|  | Substance Use Problem Severity (GAIN-SS) | 1.48 (1.21 to 1.75) | 1.53 (1.24 to 1.83) | 1.44 (1.18 to 1.70) | 1.37 (1.08 to 1.65) | 1.05 (0.81 to 1.29) | 1.52 (1.20 to 1.84) | 1.15 (0.91 to 1.39) | 1.26 (0.98 to 1.53) | 0.99 (0.76 to 1.22) | 1.13 (0.87 to 1.39) |
|  | Money spent on alcohol | 62.4 (41.0 to 83.7) | 70.5 (47.7 to 93.4) | 55.7 (31.4 to 80.0) | 75.1 (47.7 to 102.6) | 73.6 (39.4 to 107.8) | 92.1 (50.2 to 134.1) | 52.4 (28.7 to 76.0) | 76.4 (49.3 to 103.5) | 52.7 (20.2 to 85.2) | 113.7 (75.8 to 151.7) |
|  | Money spent on drugs | 157.6 (87.1 to 228.0) | 202.2 (126.1 to 278.2) | 167.0 (78.1 to 255.9) | 166.0 (61.9 to 270.2) | 146.6 (70.7 to 222.5) | 180.8 (91.8 to 269.8) | 119.2 (65.0 to 173.4) | 122.3 (62.7 to 181.8) | 136.8 (68.9 to 204.7) | 153.0 (80.0 to 225.9) |
|  | Days experiencing problems due to alcohol | 4.33 (2.92 to 5.74) | 3.40 (2.14 to 4.67) | 3.25 (1.93 to 4.56) | 4.21 (2.57 to 5.86) | 2.32 (1.22 to 3.43) | 3.59 (2.01 to 5.18) | 2.26 (1.13 to 3.39) | 3.37 (1.86 to 4.88) | 1.70 (0.82 to 2.58) | 2.86 (1.53 to 4.19) |
|  | Days experiencing problems due to drugs | 5.87 (4.27 to 7.47) | 5.15 (3.48 to 6.81) | 4.04 (2.61 to 5.48) | 5.38 (3.49 to 7.26) | 3.74 (2.34 to 5.14) | 4.46 (2.73 to 6.20) | 3.76 (2.33 to 5.19) | 3.26 (1.69 to 4.82) | 2.61 (1.42 to 3.79) | 3.83 (2.15 to 5.51) |
| Social Functioning and Quality of Life |  |  |  |  |  |  |  |  |  |  |  |
|  | Community Functioning (MCAS) | 65.3 (64.8 to 65.7) | 65.3 (64.8 to 65.8) | 67.0 (66.0 to 67.9) | 65.8 (64.8 to 66.7) | 67.0 (66.0 to 68.0) | 65.8 (64.6 to 67.0) | 68.1 (67.0 to 69.2) | 67.0 (65.7 to 68.3) | 69.1 (68.0 to 70.2) | 67.5 (66.2 to 68.7) |
|  | Physical Community Integration (CIS-PHYS) | 2.44 (2.19 to 2.69) | 2.56 (2.31 to 2.81) | 2.23 (1.96 to 2.51) | 2.21 (1.91 to 2.50) | 2.47 (2.20 to 2.74) | 2.60 (2.26 to 2.93) | 2.45 (2.18 to 2.72) | 2.62 (2.30 to 2.95) | 2.43 (2.14 to 2.72) | 2.54 (2.24 to 2.85) |
|  | Psychological Community Integration (CIS-PSYCH) | 10.6 (10.1 to 11.1) | 10.6 (10.0 to 11.2) | 12.1 (11.6 to 12.6) | 11.4 (10.9 to 12.0) | 12.3 (11.7 to 12.8) | 12.1 (11.5 to 12.7) | 12.6 (12.1 to 13.0) | 12.6 (12.0 to 13.3) | 13.0 (12.5 to 13.5) | 12.6 (12.1 to 13.2) |
|  | Quality of Life (QoLI) | 70.2 (67.1 to 73.3) | 70.4 (67.0 to 73.7) | 83.2 (80.1 to 86.3) | 78.5 (75.1 to 81.8) | 86.1 (82.9 to 89.3) | 84.8 (81.3 to 88.3) | 86.0 (82.8 to 89.1) | 83.3 (79.9 to 86.8) | 87.9 (84.8 to 91.1) | 87.0 (83.5 to 90.5) |
| Health Services Use |  |  |  |  |  |  |  |  |  |  |  |
|  | Emergency Department Visits | 1.56 (1.10 to 2.01) | 1.31 (1.06 to 1.56) | 0.85 (0.50 to 1.20) | 1.07 (0.56 to 1.57) | 0.84 (0.59 to 1.10) | 0.77 (0.47 to 1.06) | 0.77 (0.52 to 1.03) | 0.79 (0.32 to 1.26) | 0.65 (0.49 to 0.80) | 0.74 (0.35 to 1.13) |

Estimated means (95% CI) for continuous outcomes and expected counts (95% CI) were generated from models of outcome measures assessing the effect of time x treatment group, with ethnicity as a covariate. Count outcomes were limited to substance use problem severity, days experiencing problems due to alcohol, days experiencing problems due to drugs, physical community integration, emergency department visits.
