# Supplementary material for: Effectiveness of Housing First with Intensive Case Management in an Ethnically Diverse Sample of Homeless Adults with Mental Illness: A Randomized Controlled Trial
Source: PLoS One. 2015 Jul 15;10(7):e0130281. doi: 10.1371/journal.pone.0130281 (PMC4503775; doi:10.1371/journal.pone.0130281)
Supplement: S2 Table — (DOCX) [file pone.0130281.s002.docx]

**S2 Table: Treatment group differences at each study visit and changes from baseline at post-baseline visits.**

|  |  | Difference between HF-ICM and TAU groups^1^ | | | | | Change in the difference between HF-ICM and TAU from baseline^2^ | | | | | | | |
| --- | --- | --- | --- | --- | --- | --- | --- | --- | --- | --- | --- | --- | --- | --- |
|  |  | Baseline | 6 Months | 12 Months | 18 Months | 24 Months | 6 Months | | 12 Months | | 18 Months | | 24 Months | |
|  |  |  |  |  |  |  |  | *p* |  | *p* |  | *p* |  | *p* |
| Physical and Mental Health |  |  |  |  |  |  |  |  |  |  |  |  |  |  |
|  | Health Status (EQ5D-VAS) | -1.55 (-6.50 to 3.40) | 0.55 (-4.57 to 5.67) | 1.76 (-3.27 to 6.80) | -4.10 (-9.25 to 1.06) | -2.80 (-7.46 to 1.86) | 2.09 (-3.73 to 7.92) | 0.481 | 3.31 (-2.44 to 9.06) | 0.259 | -2.55 (-8.61 to 3.51) | 0.409 | -1.25 (-6.96 to 4.46) | 0.668 |
|  | Mental Illness Symptomatology (CSI) | -0.58 (-3.06 to 1.90) | -1.87 (-4.34 to 0.59) | -1.45 (-4.03 to 1.12) | -0.60 (-3.10 to 1.89) | -1.07 (-3.62 to 1.48) | -1.30 (-3.57 to 0.98) | 0.264 | -0.87 (-3.44 to 1.69) | 0.504 | -0.03 (-2.53 to 2.48) | 0.984 | -0.49 (-3.18 to 2.19) | 0.720 |
|  | Substance Use Problem Severity (GAIN-SS) | 0.97 (0.74 to 1.26) | 1.06 (0.80 to 1.40) | 0.69 (0.51 to 0.94) | 0.91 (0.68 to 1.24) | 0.88 (0.63 to 1.22) | 1.09 (0.83 to 1.44) | 0.518 | 0.72 (0.53 to 0.98) | 0.038 | 0.95 (0.69 to 1.30) | 0.737 | 0.91 (0.65 to 1.28) | 0.583 |
|  | Money spent on alcohol | -8.2 (-39.1 to 22.8) | -19.5 (-56.2 to 17.3) | -18.5 (-72.1 to 35.1) | -24.1 (-59.5 to 11.4) | -61.0 (-110.8 to -11.3) | -11.27 (-44.82 to 22.27) | 0.510 | -10.34 (-59.73 to 39.04) | 0.681 | -15.87 (-45.73 to 13.98) | 0.297 | -52.86 (-104.29 to -1.43) | 0.044 |
|  | Money spent on drugs | -44.6 (-147.7 to 58.5) | 1.0 (-137.0 to 139.0) | -34.2 (-150.0 to 81.6) | -3.1 (-82.4 to 76.3) | -16.2 (-114.3 to 82.0) | 45.58 (-105.59 to 196.75) | 0.554 | 10.39 (-121.99 to 142.78) | 0.878 | 41.53 (-73.27 to 156.33) | 0.478 | 28.42 (-96.92 to 153.76) | 0.657 |
|  | Days experiencing problems due to alcohol | 1.27 (0.77 to 2.09) | 0.77 (0.43 to 1.38) | 0.65 (0.34 to 1.24) | 0.67 (0.34 to 1.33) | 0.59 (0.29 to 1.20) | 0.61 (0.35 to 1.06) | 0.078 | 0.51 (0.28 to 0.93) | 0.029 | 0.53 (0.26 to 1.05) | 0.068 | 0.47 (0.22 to 0.99) | 0.047 |
|  | Days experiencing problems due to drugs | 1.14 (0.74 to 1.75) | 0.75 (0.46 to 1.24) | 0.84 (0.48 to 1.47) | 1.16 (0.62 to 2.16) | 0.68 (0.36 to 1.29) | 0.66 (0.39 to 1.12) | 0.123 | 0.74 (0.42 to 1.29) | 0.287 | 1.02 (0.52 to 1.98) | 0.958 | 0.60 (0.31 to 1.16) | 0.128 |
| Social Functioning and Quality of Life |  |  |  |  |  |  |  |  |  |  |  |  |  |  |
|  | Community Functioning (MCAS) | -0.04 (-0.74 to 0.67) | 1.19 (-0.15 to 2.54) | 1.17 (-0.36 to 2.71) | 1.03 (-0.66 to 2.71) | 1.64 (-0.02 to 3.30) | 1.23 (-0.13 to 2.59) | 0.076 | 1.21 (-0.33 to 2.75) | 0.122 | 1.06 (-0.64 to 2.77) | 0.222 | 1.67 (0.04 to 3.30) | 0.044 |
|  | Physical Community Integration (CIS-PHYS) | 0.95 (0.83 to 1.09) | 1.01 (0.84 to 1.22) | 0.95 (0.81 to 1.13) | 0.94 (0.79 to 1.10) | 0.96 (0.81 to 1.14) | 1.06 (0.89 to 1.27) | 0.498 | 1.00 (0.84 to 1.19) | 0.985 | 0.98 (0.83 to 1.17) | 0.843 | 1.00 (0.84 to 1.20) | 0.959 |
|  | Psychological Community Integration (CIS-PSYCH) | -0.04 (-0.80 to 0.73) | 0.69 (-0.07 to 1.45) | 0.15 (-0.66 to 0.97) | -0.10 (-0.87 to 0.68) | 0.37 (-0.43 to 1.17) | 0.73 (-0.19 to 1.65) | 0.122 | 0.19 (-0.78 to 1.15) | 0.705 | -0.06 (-1.00 to 0.88) | 0.901 | 0.40 (-0.58 to 1.38) | 0.419 |
|  | Quality of Life (QoLI) | -0.20 (-4.75 to 4.34) | 4.77 (0.18 to 9.36) | 1.29 (-3.42 to 5.99) | 2.60 (-2.03 to 7.23) | 0.92 (-3.73 to 5.57) | 4.97 (0.27 to 9.67) | 0.038 | 1.49 (-3.44 to 6.42) | 0.554 | 2.81 (-2.16 to 7.77) | 0.268 | 1.12 (-3.81 to 6.06) | 0.656 |
| Health Services Use |  |  |  |  |  |  |  |  |  |  |  |  |  |  |
|  | Emergency Department Visits | 1.19 (0.84 to 1.68) | 0.80 (0.43 to 1.48) | 1.10 (0.69 to 1.77) | 0.99 (0.51 to 1.91) | 0.88 (0.51 to 1.52) | 0.67 (0.38 to 1.19) | 0.173 | 0.93 (0.53 to 1.61) | 0.786 | 0.83 (0.42 to 1.62) | 0.585 | 0.74 (0.41 to 1.34) | 0.320 |

*P* values were assessed on the basis of the time x treatment group interaction. For continuous outcomes, the time x treatment group interaction examined the change in the mean from baseline to a subsequent follow-up visit (6-, 12-, 18- and 24-months) for the HF-ICM group compared to the TAU group and 95% CI. For count outcomes (substance use problem severity, days experiencing problems due to alcohol, days experiencing problems due to drugs, physical community integration, emergency department visits), the time x treatment group interaction evaluated the ratio of rate ratios for each post-baseline visit (e.g. rate ratio at follow-up visit relative to baseline in the HF-ICM group divided x the rate ratio at follow-up visit relative to baseline in the TAU group) and 95% CI. In these analyses, baseline values were used as a reference time point for all comparisons at subsequent time points (6-, 12-, 18- and 24-months) and the TAU group was used as a reference group.

^1^ The difference between the HF-ICM and TAU groups at each time point corresponds to mean difference (95% CI) for continuous outcomes and rate ratios (95% CI) for count outcomes (substance use problem severity, days experiencing problems due to alcohol, days experiencing problems due to drugs, physical community integration, emergency department visits) .

^2^The change from baseline to the other study time points corresponds to the mean change (95% CI) for continuous outcomes and the ratio of the rate ratios (95% CI) for count outcomes. For more details, please see table legend.
